# Takotsubo Cardiomyopathy Following Emergency Cesarean Section: A Rare Peripartum Presentation

**DOI:** 10.7759/cureus.98784

**Published:** 2025-12-09

**Authors:** Khalid A Eltayib, Khalid Ibrahim, Mohamed Youssef

**Affiliations:** 1 Department of Anesthesiology, Dubai Academic Health Corporation, Dubai, ARE; 2 Department of Radiology, Dubai Academic Health Corporation, Dubai, ARE

**Keywords:** cesarean section, obstetric anesthesia, peripartum cardiac event, stress-induced cardiomyopathy, takotsubo cardiomyopathy

## Abstract

Takotsubo cardiomyopathy (TCM), also known as stress-induced cardiomyopathy or transient left ventricular ballooning, presents with suspicion of an acute myocardial infarction characterized by temporary apical or midventricular dyskinesia of the left ventricle, despite normal findings on coronary angiography. This temporary cardiac condition is frequently triggered by episodes of emotional or physical stress. We present an unusual case of TCM occurring immediately after an emergency cesarean section in a 41-year-old woman diagnosed with gestational diabetes. The patient experienced acute respiratory distress and chest discomfort soon after being extubated, necessitating reintubation and intensive care unit management. Electrocardiogram showed nonspecific ST-T changes and mild ST elevation in V1-V2, and echocardiogram results indicated apical and midventricular akinesia along with a decreased ejection fraction, consistent with TCM diagnosis. A subsequent coronary angiography displayed normal coronary arteries. The patient was treated conservatively with supportive measures and cardiac medications. This case report underscores the importance of identifying stress-induced cardiomyopathy during the peripartum period, particularly following high-stress obstetric procedures.

## Introduction

Takotsubo cardiomyopathy (TCM), often referred to as stress-induced cardiomyopathy or "broken heart syndrome," is a temporary heart condition marked by sudden left ventricular dysfunction without the presence of obstructive coronary artery disease. Initially identified in Japan during the 1990s, the syndrome is named after the traditional Japanese octopus trap ("takotsubo") because of the typical apical ballooning observed on imaging studies during the systolic phase [1].

Although TCM predominantly affects postmenopausal women, its occurrence in the peripartum period is increasingly recognized, albeit still rare. Peripartum cardiomyopathy (PPCM) is defined as heart failure related to pregnancy, characterized by a left ventricular ejection fraction of less than 45%. PPCM arises during the peripartum period and does not represent the worsening of any form of cardiomyopathy that existed before pregnancy [2].

The underlying mechanism is thought to involve an increase in catecholamines caused by physical or emotional stress, resulting in myocardial stunning and temporary systolic dysfunction [3]. Pregnancy and delivery, particularly cesarean sections, represent significant physiological and psychological stressors that may precipitate TCM in susceptible individuals [4].

Several case reports and reviews have documented TCM in the context of pregnancy, with most cases occurring in the immediate postpartum period. However, its presentation during or immediately following cesarean section remains uncommon and diagnostically challenging due to overlapping features with other peripartum cardiac conditions such as PPCM [5,6]. Timely identification is essential, as the clinical indicators frequently resemble those of acute coronary syndrome, presenting with symptoms like chest discomfort, shortness of breath, alterations in electrocardiogram (ECG), and increased levels of cardiac biomarkers [7].

This case report describes a rare instance of TCM following an emergency cesarean section, highlighting the importance of considering TCM in the differential diagnosis of acute cardiac events in the peripartum period. We also discuss the diagnostic approach, management strategies, and outcomes in the context of current literature.

## Case presentation

A 41-year-old gravida 5 para 4 woman at full term presented in labor. Her obstetric history included four previous vaginal deliveries. She had gestational diabetes managed with metformin 750 mg once daily and dietary control. There was no history of cardiovascular disease, surgical procedures, or prior anesthesia exposure.

After doing a preoperative anesthesia evaluation and obtaining anesthesia consent, due to severe fetal bradycardia, an emergency cesarean section was performed under general anesthesia. Induction involved propofol 150 mg, lidocaine 70 mg, and rocuronium 50 mg, followed by smooth intubation with a 7 mm endotracheal tube. The procedure was uneventful, lasting 45 minutes, with an estimated blood loss of 500 mL and a positive fluid balance of +780 mL. Postdelivery analgesia included fentanyl 100 mcg, parecoxib 40 mg, and paracetamol 1,000 mg. Uterotonic agents administered included carbetocin, methergine, and oxytocin infusion.

**Figure 1:**
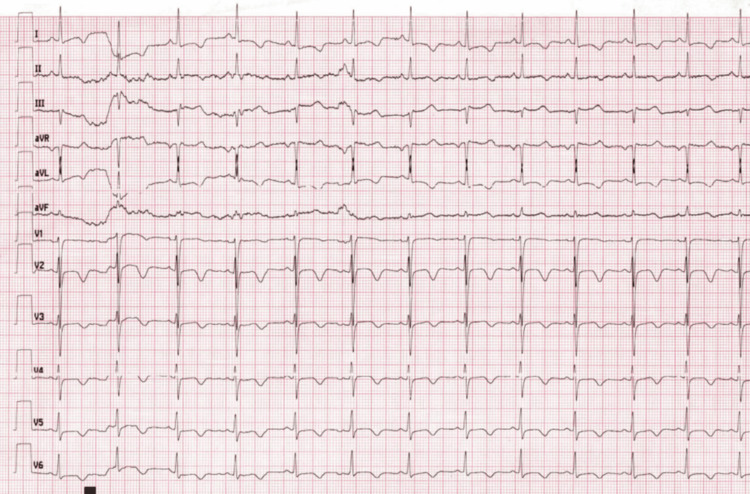
Initial electrocardiography, showing sinus rhythm with nonspecific ST-T changes and mild ST elevation in V1-V2 aVR: augmented vector right; aVL: augmented vector left; aVF: augmented vector foot

Toward the end of the procedure, the patient developed sinus tachycardia (HR, 160-170 bpm) with elevated blood pressure. Morphine 10 mg IV was administered for analgesia. After extubation, the patient became irritable and dyspneic, reporting chest tightness. Oxygen saturation dropped to 75%, prompting immediate reintubation and ventilation with 100% oxygen. Initial ECG showed sinus rhythm with nonspecific ST-T changes and mild ST elevation in V1-V2 (Figure 1).

**Figure 2:**
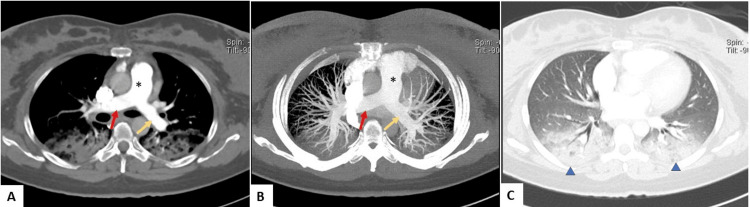
CT imaging: pulmonary angiography. (A) Axial pulmonary angiogram. (B) Axial maximum intensity projection. (C) Axial lung window Normal opacification of the main pulmonary trunk (asterisk), right pulmonary artery (red arrow), and left pulmonary artery (yellow arrow), showing no pulmonary embolism. Bilateral basal pulmonary densities mostly represent postanesthesia atelectasis (blue arrowheads)

CT pulmonary angiography was performed to exclude pulmonary embolism and was negative (Figure 2). The patient was transferred to the ICU for further evaluation.

Venous blood gas analysis revealed respiratory acidosis, hypoxemia, and elevated lactate. Cardiac enzymes showed elevated Troponin level with borderline increase in creatine kinase-myocardial band and B-type natriuretic peptide and D-dimer tests (Table 1).

**Table 1:** Cardiac markers on day 1 Elevated cardiac markers are visible CK-MB: creatine kinase-myocardial band; NT-proBNP: N-terminal-pro-BNP; FEU: fibrinogen equivalent units

Lab test	Result	Interpretation	Normal range
Troponin T	222	High	<14 ng/L
CK-MB	6.1	High	<4.89 ng/mL
NT-proBNP	260.8	High	<125 pg/mL
Dimer test	2.66	High	<0.5 ug/mL FEU

**Figure 3:**
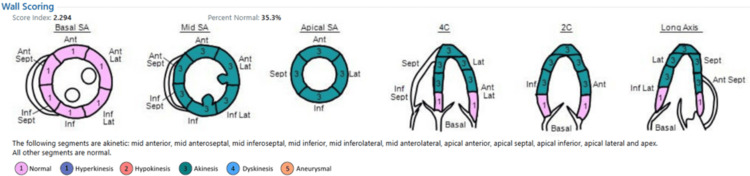
The first echocardiogram immediately after the event, showing apical and mid-SA, with normal basal segments SA: segment akinesia

Differential diagnoses included 1) TCM, 2) acute coronary syndrome, and 3) acute pulmonary embolism. Bedside echocardiography demonstrated apical and mid-ventricular akinesia with preserved basal segments, an ejection fraction of 27%, moderate mitral regurgitation, and mild tricuspid regurgitation, findings consistent with TCM (Figure 3).

**Figure 4:**
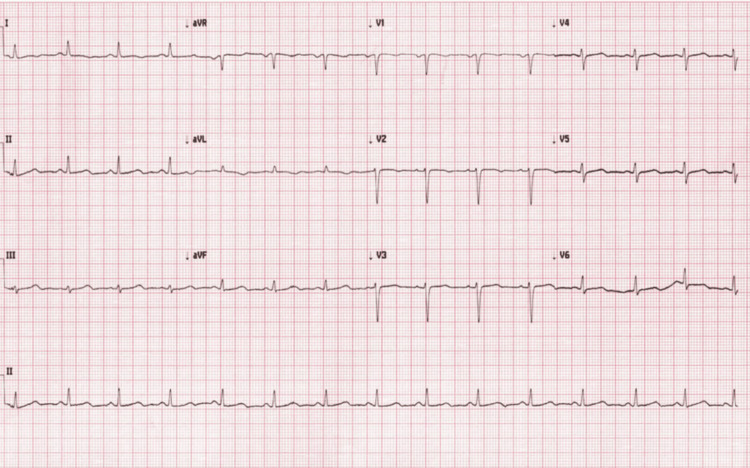
Second day electrocardiography, showing regression of the ST segment elevation and ST-T changes aVR: augmented vector right; aVL: augmented vector left; aVF: augmented vector foot

Coronary angiography was deferred as per cardiology consultation. The patient was started on valsartan 40 mg and bisoprolol 2.5 mg, with full respiratory support. On the second day, the patient was extubated, and her clinical condition improved. The ECG was repeated, showing regression of the ST-segment elevation and ST-T changes (Figure 4).

**Figure 5:**
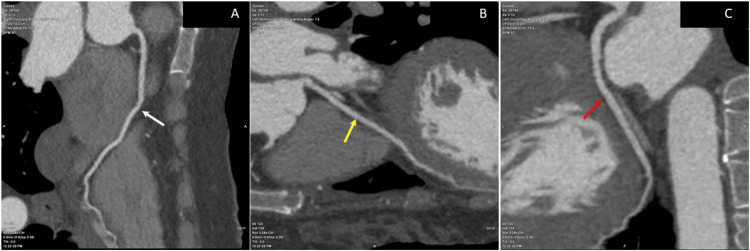
Processed images of a CT cardiac angiogram, showing normal coronary arteries outline and course, and no evidence of atherosclerotic coronary disease. (A) Right coronary artery. (B) Left anterior descending artery. (C) Left circumflex artery

Then, the cardiology workup was done, including a CT cardiac angiogram, which showed normal coronary arteries (Figure 5), supporting the diagnosis of TCM.

**Figure 6:**
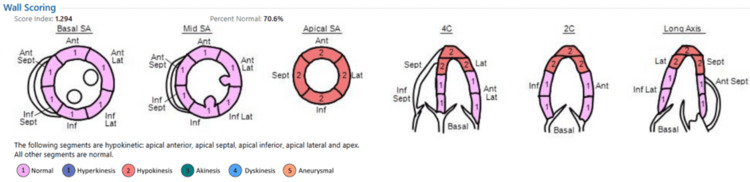
The second echocardiogram done one week after the event, showing significant improvement in wall motion abnormalities SA: segment akinesia

Echocardiography was repeated before discharge from the hospital on the sixth postoperative day, which showed marked improvement in wall motion abnormalities described on the first study with an ejection fraction of 44% (Figure 6). The patient made an excellent recovery thereafter.

## Discussion

TCM is a reversible cardiac condition often triggered by sudden stress. In obstetric patients, factors such as emotional stress, changes in hemodynamics, and medications like methergine may trigger this condition. This case demonstrates the development of TCM immediately after a cesarean section, likely due to a combination of surgical stress, anesthesia, and hemodynamic changes around delivery [7,8]. Minatoguchi et al. conducted a review of 18 pertinent cases of peripartum TCM from existing literature. Among these, 16 cases occurred after delivery, and two cases occurred during pregnancy. The majority of the women (81%) had cesarean deliveries, and the first signs of TCM were reported during surgery in 38% of the instances. The most common symptoms included chest pain (44%) and difficulty breathing (28%). Nearly all cases (94%) showed abnormalities on the ECG, such as ST-segment changes and T-wave inversion. Elevated serum levels of cardiac enzymes were found in 92% of the cases [4]. Follow-up echocardiography revealed that left ventricular systolic function had returned to normal within six months for all cases. This case report and review highlight that TCM may be masked in postpartum women by symptoms that mimic those of acute coronary syndrome, PPCM, or pulmonary embolism, suggesting that echocardiography may be an effective diagnostic tool for differentiating among these conditions [9]. Ruiz et al. reviewed 20 cases of TCM; 17 occurred during the postpartum period, whereas three occurred during pregnancy. Five of these cases involved vaginal births, while the rest were cesarean sections, with five cases manifesting intraoperatively. Aside from one patient with normal ECG findings, all other patients exhibited ECG changes and elevated cardiac enzyme levels. Nonetheless, cardiac catheterization showed no coronary artery damage in any of the cases. After three months, all patients had fully recovered [10].

The clinical presentation resembled that of pulmonary embolism and acute coronary syndrome, highlighting the necessity for a comprehensive differential diagnosis. Echocardiography was crucial in recognizing the typical wall motion abnormalities associated with TCM. The primary approach to management is supportive, with the use of beta-blockers and angiotensin-converting enzyme inhibitors facilitating recovery [11]. Recovery of cardiac function is frequently observed within a month, though some individuals may need ongoing heart failure treatment [7]. Citro et al. reported a case of temporary left ventricular ballooning that occurred shortly after childbirth following an ergonovine injection [12], which progressed quickly from "typical apical" ballooning to a "midventricular" myocardial failure, drawing attention to the sympathomimetic effects of commonly used uterotonics.

This case report contributes to the sparse literature on peripartum TCM and underscores the importance of increased awareness among obstetricians, anesthesiologists, and intensivists.

## Conclusions

TCM should be considered in postpartum patients who show acute cardiac symptoms, particularly after high-stress deliveries. Prompt identification and supportive care are crucial for achieving positive outcomes. Teamwork across various specialties is important when dealing with these complex cases. TCM should be considered in postpartum patients who show acute cardiac symptoms, particularly after high-stress deliveries. Prompt identification and supportive care are crucial for achieving positive outcomes. Teamwork across various specialties is important when dealing with these complex cases.
